# Pneumonia in rural Malawians under five years old: Treatment outcomes and clinical predictors of death on admission

**DOI:** 10.4102/phcfm.v1i1.43

**Published:** 2009-09-01

**Authors:** Prosper M. Lutala, Suzgo Mzumara, Maurice Mlenga, Raphael Talipu, Eric Kasagila

**Affiliations:** 1 Department of Family Medicine, University of Goma, Democratic Republic of Congo; 2 Mchinji District Health Office, Malawi; 3 Department of Community Medicine, College of Medicine, University Of Malawi

**Keywords:** pneumonia, children, Malawi, death, risk factors

## Abstract

**Background:**

High mortality and disability due to pneumonia occur worldwide. The introduction of the Integrated Management of Childhood Illness strategy in Malawi brought with it hope of an improvement in the outcome of pneumonia. However, the risk of death and treatment outcomes remain unknown in many districts.

**Method:**

The medical records of 466 consecutive patients admitted to the Mchinji District Hospital from January 2004 to January 2006 whose disease met the World Health Organization criteria for pneumonia were reviewed. Data were collected from forms that had been filled out and different treatment outcomes and determinants of death were analysed using logistic regression.

**Results:**

Of the 466 patients, 62.7% completed treatment, 15.9% had unknown outcomes, 12.9% died, 8.4% were lost to follow-up, 0.8% failed to improve with treatment, and 0.4% were transferred to other facilities. Independent predictors of death were: age less than 2 years, female sex, history of pneumonia, chest retractions, type of pneumonia, and central cyanosis.

**Conclusion:**

A high proportion of deaths and unknown outcomes occurred among participants. Young age, female sex, history of pneumonia, chest retractions and central cyanosis were associated with death. Mortality from pneumonia may be reduced by close monitoring of these risk factors and by improving health education programmes and communicating these findings to parents and health workers. Further investigations of local reasons for high rates of unknown/unreported outcomes are welcomed.

## INTRODUCTION

Between 1960 and 1990, child mortality in developing regions was halved to 1 child in 10 dying before the age of five years. Six causes account for 73% of the 10.6 million deaths in this population: pneumonia, diarrhoea, malaria, neonatal sepsis, preterm delivery and asphyxia at birth.^1^ An estimated 1.9 million children die each year due to acute respiratory infections (ARIs), mainly pneumonia. According to a report by the World Health Organization (WHO), about 3.9 million deaths and the loss of 94.6 million disability-adjusted life years occur worldwide due to respiratory infections alone.^2^

The Malawi National Health Information System listed respiratory illness as the second most common outpatient disease after malaria in children under five years of age, constituting 18.2% of all outpatient conditions. Respiratory illness was the third main inpatient disease, representing 10.1% of admissions and accounting for 8.8% of the case-fatality rate.^3^

Malawi's gross domestic product (GDP) was only $1.8 billion in 2003, with around 60% of the population living below the poverty line. This poverty is a result of a combination of several factors including: underdeveloped institutions; poor physical infrastructure; corruption; low human capital investment reflected in limited educational provision; undiversified exports; difficult climatic conditions; an adverse geographical position restricting access to international markets; and relatively few natural resources.^4^ Large proportions of the population require food aid, with substantial numbers of malnourished children in feeding centre programs.^5^ Malawi's economy and public services are further weakened by long-term morbidity among people in their productive age and by an estimated 80 000 premature deaths due to HIV each year^6^.

In rural Malawi, as in many rural settings in Sub-Saharan Africa, solid fuels remain the principal household source of energy for cooking, heating and lighting. Biomass fuels that are used include plant or animal materials such as wood, charcoal and dung residues. These account for more than one half of domestic energy. People live in small houses with a lack of sufficient ventilation and poorly designed stoves that do not have flues or hoods to remove smoke from living areas.^7, 8^ The combination of the above-mentioned factors with incomplete immunisation and late care-seeking behaviour drastically increases the likelihood of severe pneumonia.^7–9^

The Mchinji district is populated by about 456 314 inhabitants including 77 573 under-fives. The district is served by a 180-bed district hospital.

Pneumonia case management has been well implemented in Malawi by means of the Integrated Management of Childhood Illness (IMCI) strategy and led to a 29% decrease in mortality among children under five years of age between 2000 and 2006.^10^ Yet, child mortality due to pneumonia could probably be reduced further by using a combination of approaches.^11–14^ Despite studies assessing the state of pneumonia management related to the above domains, nothing has yet been done to address these issues in the Mchinji district. Therefore, the objectives of the study were to determine, firstly, which risk factors best predict death from pneumonia at the Mchinji District Hospital, and secondly, the outcomes of treatment using the national syndromic guidelines for pneumonia treatment in children under five years old.

## METHOD

A retrospective study was carried out in the paediatric ward of Mchinji District Hospital, rural Malawi.

All children consecutively admitted to the ward from January 2004 to January 2006 were included in the study. The data were collected from a special form called the ‘yellow card’ or ‘Pneumonia Inpatient Recording Form’ (see Figure 3) and were cross-checked with information from pneumonia registries covering the study period. For the purpose of this study, the type of pneumonia was classified according to the WHO definition.^15^ The treatment was decided on according to the severity of the pneumonia, in line with IMCI guidelines. Oral co-trimoxazole was prescribed for simple pneumonia, benzylpenicillin for severe pneumonia, and chloramphenicol for very severe pneumonia. For infants less than two months of age, both benzylpenicillin and gentamycin were given. Within four days following hospital discharge for children two months to five years old (and within five days for newborn babies), the regimen was switched to amoxicillin to complete the course of treatment. The prescription of oral amoxicillin was initiated once all the clinical features of severe and very severe pneumonia from simple pneumonia had subsided. Upon discharge, the next of kin/mother was advised to take the child to the health facility for follow-up after completing the course of antibiotics, to ascertain healing and to record the final outcome.

Apart from death, the treatment outcomes were divided into five groups: (1) treatment completed (course of antibiotics completed and/or full recovery, meaning no clinical features persisted and the patient returned to his/her initial state of health following a successful course of treatment); (2) failure at 48 hours (worsening of rapid breathing, worsening of inspiratory retractions, development/persistence of abnormal sleepiness or difficulty in awakening, or development/persistence of inability to drink or poor breastfeeding); (3) self-discharge against medical advice (child removed from the hospital against medical advice before treatment was completed), (4) loss to follow-up (the child was referred for treatment to another health facility and the result of treatment in this second facility was unknown; when the result was known, it should have been recorded in place of the result ‘transferred’); (5) outcome unknown (when the parent and child did not return for a follow-up visit after completion of the intensive phase of antibiotic(s) and the patient was not discharged by a health professional). Respiratory rates were divided into normal and abnormal, depending on the age of the child. For children 12 months old or under, a high respiratory rate was more than 60 breaths per minute (HRR_1_); for those between 12 and 36 months, more than 40 breaths per minute (HRR_2_); and for those between 36 and 59 months, more than 34 breaths per minute.^16^ Age range was stratified in 3 groups (A_1_: 0–2 months; A_2_: 2–24 months; and A_3_: 24–59 months).

Central cyanosis was defined as oxygen desaturation, as opposed to venous blood. It is translated by blue discolouration of lips and nail beds and cyanosis of mucus and skin, and is measured using a pulse oximetry.^17^

Data were captured using STATA version 9.0 for Microsoft Windows.^18^ Multivariable logistic regression was performed with pneumonia death or survival as the outcome. All confounding factors (treatment options, age ranges, and types of pneumonia) were entered and adjusted to investigate their contribution to mortality. Since the number of patients with ‘unknown outcomes’ and ‘[loss] to follow-up’ were high, it was decided to perform a sensitivity analysis with best- and worst-case scenario testing. This was done by assuming the best outcome, i.e., ‘survival’ for all patients with unknown outcomes or loss to follow-up (best case), and then assuming the worst outcome, i.e., death, for these same patients (worst case). Also, the treatment options in the acute phase were introduced into the model as an independent factor to discover their predictors of death. All variables that were associated with death at the significance level of P < 0.10 in the univariate analysis were included in the initial model. The significance level for removal from the model was set at P = 0.06 and that for addition to the model at P = 0.05. When appropriate, 95% confidence intervals (CIs) were calculated for the proportions.

## RESULTS

Table 1 summarises the demographic and baseline clinical characteristics of the patients. Most patients were female and had severe pneumonia, and the highest proportion of patients was admitted in 2005.

**TABLE 1:** Demographic and baseline clinical characteristics of patients

VARIABLE	NUMBER OF CHILDREN	PERCENTAGE	95% CONFIDENCE INTERVAL
**Sex**			
Male	207	44.4	
Female	257	55.2	
Missing values	2	0.4	
**Type of pneumonia**			
Simple pneumonia	27	5.8	4.0–8.3
Severe pneumonia	257	55.2	50.6–59.6
Very severe pneumonia	171	36.7	32.4–41.2
Missing values	11	2.4	1.3–4.2
**Year of admission**			
2004	180	38.6	34.3–43.1
2005	264	56.7	52.1–61.1
2006	18	3.9	2.5–6.0
Missing values	4	0.8	0.3–2.2

**TOTAL**	**466**	**100**	

### Treatment outcomes

**FIGURE 1:**
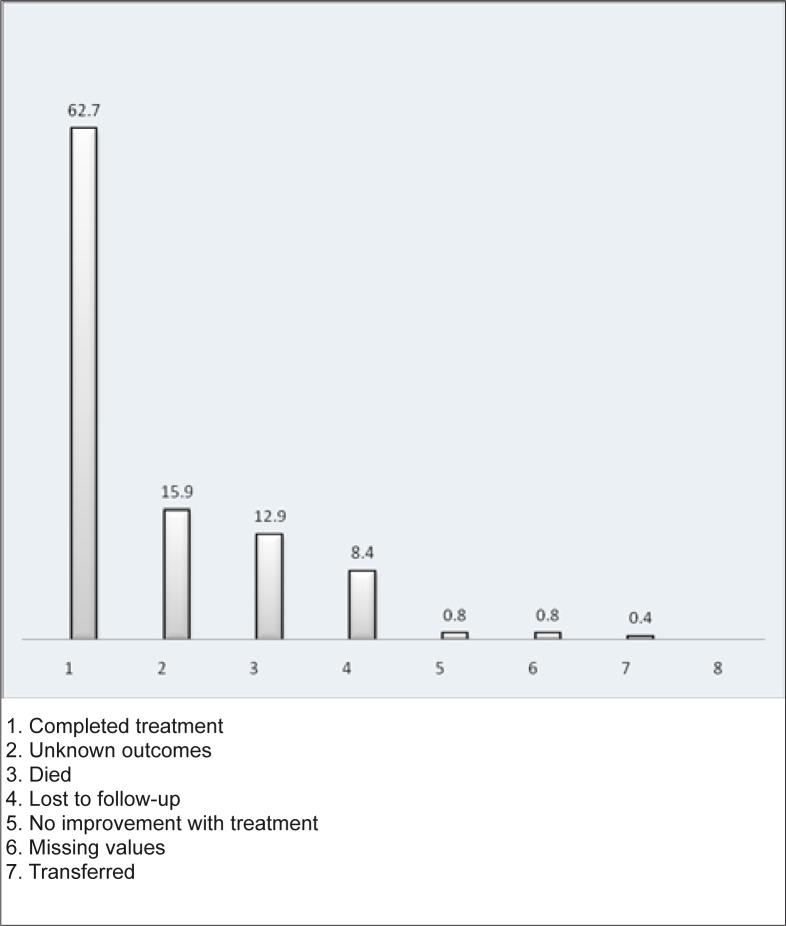
Different outcomes of treatment

Figure 1 summarises the treatment outcomes and demonstrates that the most common outcome was completion of treatment (62.7% of patients), followed by unknown outcome (15.9%).

**FIGURE 2:**
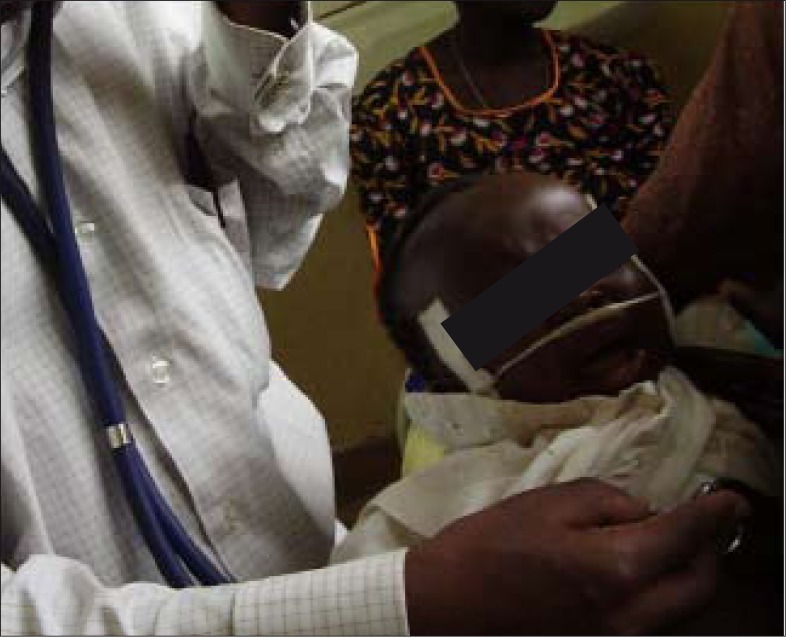
Child with severe pneumonia in Mchinji Hospital

**FIGURE 3:**
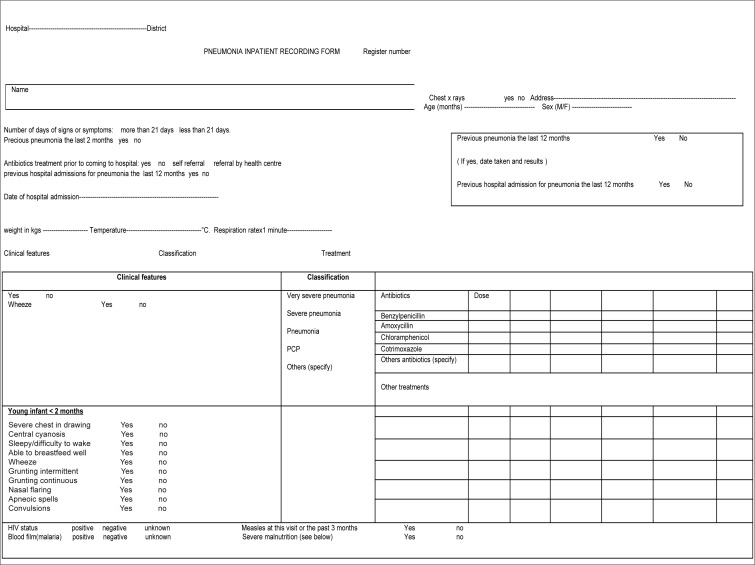
Pneumonia inpatient recording

### Mortality risk factors

In Table 2 the best case scenario was assumed, and in Table 3, the worst case scenario. Female sex, history of pneumonia, type of pneumonia (i.e. severity), chest in-drawing/ chest retractions, and central cyanosis (Table 2) were independently associated with death. For each variable, except type of pneumonia, the odds ratios for death were similar for the best and worst case scenarios. Therefore, according to the analysis, missing information about patients with unknown outcomes did not significantly affect the risk that was ascribed to each variable, except the type ‘very severe pneumonia’.

**TABLE 2:** Risk factors independently associated with death (best case scenario: All patients with unknown outcomes assumed to have survived/recovered)

VARIABLES	CRUDE VALUES		ADJUSTED VALUES	
	
	OR (95% CI)[1]	P–VALUE	OR (95% CI)[2]	P–VALUE
Age 0–2 months	0.96 (0.93–0.99)	0.01	0.96 (0.93–0.99)	0.04
Sex	1.11 (0.65–1.92)		0.7	
Female sex	0.91 (0.25–0.99)		1.48 (1.05– 4.05)	0.02
History of pneumonia	1.13 (0.46–2.79)	0.78		
PAP[3]	0.66 (0.33–1.44)	0.29		
Respiratory rate	1.01 (0.99–1.04)	0.24		
Inspiratory chest retractions	0.32 (0.11–0.92)	0.03	0.37 (0.12–1.10)	0.07
DDF[4]	0.55 (0.32–0.95)	0.03	0.59 (0.33–1.07)	0.08
Central cyanosis	0.21 (0.10–0.42)	0	0.32 (0.15–0.67)	0
Major signs presents	1.06 (01.0–1.11)	0.05	1.03 (0.97–1.09)	0.31
Type of pneumonia	2.01 (1.13–3.07)	0	1.75 (1.00–3.06)	0
very severe	5.4 (2.89–10.1)	0	5.70 (3.04–13.21)	0.02
Associated malaria	0.68 (0.38–1.19)	0.18		
Antibiotic treatment	0.93 (0.84–1.03)	0.15		
Additional treatment	0.70 (0.40–1.19)	0.19		
Year of admission	0.73 (0.54–0.99)	0.04	0.93 (0.64–1.34)	0.69
Dry/cold season (May– August)	0.99 (0.94–1.03)	0.58		

**TABLE 3:** Risk factors undependably associated with death (worst case scenario: All patients with unknown outcomes assumed to have died)

VARIABLES	CRUDE VALUES		ADJUSTED VALUES	
	
	OR (95% CI)	P–VALUE	OR (95% CI)	P–VALUE
Age	0.9 (0.97–1.01)	0.5		
Sex	1.1 (0.79–1.68)	0.4		
History of pneumonia	0.7 (0.4–1.3)	0.3		
PAP	0.6 (0.3–1.2)	0.22		
Respiratory rate	1.0 (0.9–1.0)	0.5		
Inspiratory chest retractions	0.58 (0.34–0.99)	0.05	0.61 (0.35–1.05)	0.07
DDF	1.11 (0.75–1.63)	0.61		
Central cyanosis	0.48 (0.25–0.91)	0.03	0.51 (0.26–0.97)	0.04
Major signs presents	1.02 (0.98–1.06)	0.43		
Type of pneumonia	1.17 (0.86–1.60)	0.32		
Very severe	1.69 (1.13–2.51)	0.01	1.54 (1.01–2.33)	0.04
Associated malaria	0.85 (0.56–1.28)	0.43		
Antibiotic treatment	0.98 (0.92–1.04)	0.6		
Additional treatment	0.75 (0.51–1.09)	0.13		
Year of admission	0.87 (0.71–1.06)	0.17		
Cold season	1.03 (0.99–1.06)	0.1		

Stratification of ages did not show any difference between the best versus worst case scenarios: Age_1_ (1.52 [95% CI 0.59–2.96]; P: 0.50) versus (1.04 [95% CI 0.57–1.90]; P: 0.81), Age_2_ (1.07 [95% CI 0.57–2.04]; P: 0.22) versus (OR1 [95% CI 0.65–1.56]; P: 0.02), Age_3_ (0.67 [95% CI 0.28–1.64]; P: 0.38) versus (0.96 [95% CI 0.56–1.67]; P: 0.89).

The same was found for respiratory rates among the groups: in RR_1_(1.46 [95% CI 0.81–2.66]; P: 0.21) versus (1.27 [95% CI 0.83– 1.95]; P: 0.28), RR_2_(1.76 [95% CI 0.40-7.68]; P: 0.45) versus (1.96 [0.45–2.45]; P: 0.90).

Male sex did not reveal any differences relating to death risk from pneumonia in both scenarios: (0.90 [95% CI 0.52–1.55]; P: 0.70) in first scenario versus (0.87 [0.59–1.27]; P: 0.87) in the second.

An analysis of the probability of survival associated with different antibiotic regimens (Table 4) was conducted due to fact that the ineffectiveness of some antibiotics used in pneumonia regimens to prevent death was presumed. Treatment with chloramphenicol was associated with survival (OR 5.6 [95% CI, 3.2–10.6]) when used alone. In contrast, benzyl penicillin used on its own was associated with the lowest probability of survival (OR 0.26 [95% CI 0.13–0.53]) in the best scenario.

**TABLE 4:** Treatment option as independent variable in prediction of survival in the best and worse scenarios

VARIABLE	BEST SCENARIO		WORST SCENARIO	
	
	OR (95% CI)	P–VALUES	OR (95%CI)	P–VALUES
Benzyl penicillin (BP)	0.3 (0.13–0.53)	0	0.7 (0.46–1)	0.05
Chloramphenicol (cc)	5.6 (3.19–10.6)	0	1.9 (1.32–3.0)	0
BP + gentamycin (gen)	2.2 (0.23–21.8)	0.49	1.2 (0.56–2.6)	0.62
cc+ gentamycin	6.7 (0.41–109)	0.18	1.7 (0.11–27)	0.71
BP + chloramphenicol	1.4 (0.40–5.17)	0.57	0.9 (0.33–2.5)	0.87

## DISCUSSION

The results show that the mortality in this study (12.9%) was higher than the national case-fatality rate previously reported (8.85%). This mortality may be an underestimate of the actual mortality due to several reasons. Firstly, the high prevalence of HIV in the country with a high mortality of pneumonia due to *Pneumocystis jirovecii* in Malawian children has been described elsewhere.^19^ Secondly, HIV-infected children, especially infants, with severe pneumonia fail WHO-standard treatment with parenteral penicillin or amoxicillin more often than do HIV-negative children^20^ and a high mortality of pneumonia-HIV co-infection is well known.^21^ Thirdly, rates of resistance to cotrimoxazole are very high, particularly in Sub-Saharan countries like Malawi where cotrimoxazole is used to prevent pneumonia,^22^ malaria, and intestinal and cerebral infections. In some guidelines, amoxicillin is now preferred as a first-line treatment for pneumonia, based in part on the finding that more severe cases do better with amoxicillin.^23^ Despite this, cotrimoxazole remains the recommended drug used in cases of simple pneumonia in Malawi in view of its efficacy, cost, and ease of use.^2, 25^ The risk of survival increased almost six-fold (Table 4) once chloramphenicol had been administered, but was relatively unaffected when penicillin was given. This finding may reflect the development of some bacterial strains that are resistant to penicillin or lack of stratification between severe and very severe pneumonia; two forms with different prognoses. The overall case-fatality rate reported at the Queen Elizabeth Central Hospital in Blantyre, Malawi was 22%,^16^ almost double the rate we found (12.9%). This difference can be explained by the higher prevalence of HIV/AIDS in urban, densely populated cities of Malawi such as Blantyre, as opposed to the rural areas. In addition, a few cases of death were possibly overlooked in our study due to the high rate of unknown outcomes and transfers without follow-up data. The Blantyre study reported the mortality rate of severe cases while this study included all three categories. The case-fatality rate of this study is higher than the rates found in Pakistan^26^ before and after the introduction in 1996 of the IMCI case management of pneumonia (9.9% and 4.9% respectively).

The treatment outcomes seemed satisfactory in 62.3% of patients who completed the course of antibiotics (Figure 1). However, the rate of patients lost to follow-up or with unknown outcomes constituted 15.9% and 8.4% respectively; this being of great concern. In fact, these two figures can provide insight into the actual conditions of the health care system. Several possible factors may explain these relatively high numbers: referral system dysfunction; lacking negotiation skills on the part of those providing counselling on discharge; insufficient numbers of trained auxiliary staff in the community to liaise with the hospital; lack of strong emphasis on community pneumonia management in the guidelines; and social attitudes in African culture that tend to give little importance to prevention.

The results of this study are in line with previous studies which showed that moderate to severe alteration of general health status,^26^ inspiratory retractions, and age between 2 and 11 months^26, 27^ were significantly associated with death. Another death and predictors of different treatment outcomes have been study^18^ showed, contrary to the findings of this study, that cyanosis identified. Close monitoring of identified risk factors in children and poor feeding were strong predictors of death compared to admitted for pneumonia could reduce the burden of disease in other variables. However, in areas such as Malawi with patients this hospital. Furthermore, knowledge of the risk factors may be from highly pigmented race groups, where moderate and severe used to improve the efficiency of health education programmes anaemia (Hb < 7g/dl) are common, particular difficulties are for parents and health workers aimed at reducing pneumonia found in using cyanosis as a clinical sign of hypoxaemia.^17^

### Limitations of the study

The main limitations of this study result from its retrospective nature. Data on many patients were not available. More importantly, the lack of information on variables like HIV status and nutrition compromised the analysis of predictors of death, even though questions are asked about these variables on the ‘yellow card’ or Pneumonia Inpatient Recording Form designed by the Malawian government. Finally, the study was limited by the large number of patients who were lost to follow-up due to the reasons described above. One of the strengths of the study was the inclusion of different seasons during consecutive years; data was validated by checking two different sources. Also, the results were validated by minimising the effect of the high number of unknown outcomes on the determination of risk factors for death by conducting analyses based on two alternatives scenarios.

### Conclusion

The case management of pneumonia in paediatrics in the Mchinji District Hospital is following the standard of care prescribed by the WHO successfully. However, a high case-fatality rate and a high number of unknown outcomes persist. Risk factors for death and predictors of different treatment outcomes have been identified. Close monitoring of identified risk factors in children admitted for pneumonia could reduce the burden of disease in this hospital. Furthermore, knowledge of the risk factors may be used to improve the efficiency of health education programmes for parents and health workers aimed at reducing pneumonia mortality. Studies are warranted to further elucidate and address the reason why so many children with pneumonia are lost to follow-up or have ‘unknown outcomes’.
